# Thyroidectomy in children and adolescents: distinguishing benign from malignant thyroid nodules

**DOI:** 10.1016/j.jped.2025.101426

**Published:** 2025-08-21

**Authors:** Mário Sérgio Rocha Macedo, Jônatas Catunda de Freitas, Débora Cabral Coutinho, Carina Marques Barroso, Sandra Regina Geroldo, Rhuce Pedrosa Carvalho, Letícia Siqueira Monte Alverne, Tamyres Ferreira Campos, Lívia Rolim Fernades Macedo, José Huygens Parente Garcia, Wellington Alves Filho

**Affiliations:** a Hospital Infantil Albert Sabin, Fortaleza, CE, Brazil; b Universidade Federal do Ceará, Departamento de Cirurgia, Fortaleza, CE, Brazil; c Unicristhus, Fortaleza, CE, Brazil

**Keywords:** Thyroidectomy, Children, Nodule

## Abstract

**Objectives:**

The research aimed to analyze the clinical, epidemiological, laboratory, imaging, and pathological characteristics of pediatric patients who underwent surgery for thyroid nodules, distinguishing and comparing malignant and benign conditions.

**Methodology:**

A retrospective and descriptive analysis was conducted on patients aged 0–18 years who underwent thyroidectomy. The following variables were evaluated: gender, previous and current pathological history, laboratory thyroid hormone levels, ultrasonography, cytology, and histopathological findings.

**Results:**

A total of 91 children underwent thyroidectomy from January 2013 to May 2023. The average age was 13 years, and 68 patients were female. Ultrasonography evaluation revealed that 62 children had unimodular disease. Histopathology reports showed benign tumors in 54 patients and malignant tumors in 37. The factors associated with a higher incidence of cancer in univariate analysis were age < 10 years (*p* = 0.025), the presence of nodules on ultrasound with microcalcification (*p* < 0.001), and hypoechogenic nodules (*p* = 0.007). On multivariate analysis, only age under 10 years (OR = 5.69; *p* = 0.028) and the presence of microcalcifications (OR = 28.44 and *p* = 0.002) were significantly associated with malignancy.

**Conclusion:**

The medical community is becoming increasingly aware of the differences in the clinical, epidemiological, pathological, and molecular presentations of benign and malignant thyroid diseases, highlighting the need for further training in this area.

## Introduction

For years, physicians, particularly pediatricians, endocrinologists, and head and neck surgeons managing thyroid pathologies in pediatric patients, have relied on limited and occasionally anecdotal evidence in the literature to guide their clinical approaches. In 2009, the American Thyroid Association (ATA) recommended that the diagnostic and therapeutic approach to one or more thyroid nodules in children should be the same as in adults.^1^ Later, in 2015, with better knowledge of clinical, pathological, and molecular differences between these two groups, the ATA published its first guideline on the management of thyroid nodules and well-differentiated thyroid cancer for the pediatric public.^2^ Then, in 2022, the European Thyroid Association (ETA) and the Multidisciplinary Guideline Development Group (UK) emphasized the promotion of best practice standards for the diagnosis and treatment of nodules and well-differentiated thyroid cancer for the pediatric population.^3^,^4^

As in adults, thyroid cancer in children has increased in recent decades, leading to a greater number of pediatric patients undergoing thyroid surgery.^5^,^6^ In addition to malignant conditions, various benign disorders, including large nodular goiters, hyperthyroidism caused by autonomous adenomas, Graves' disease refractory to medical management, and prophylactic indications for familial medullary carcinoma, may indicate total or partial thyroidectomy.^7^,^8^ Thus, greater awareness of this disease, the creation of specialized centers with experienced teams in the management and follow-up of children with thyroid pathologies, as well as the formation of collaborative networks between these centers are important steps in achieving better results.^9^

The study aimed to evaluate the clinical, epidemiological, laboratory, imaging, and pathological characteristics of pediatric patients who underwent surgery for thyroid nodules, with a focus on distinguishing and comparing malignant and benign conditions.

## Methodology

This single-center retrospective and descriptive study was conducted at a comprehensive pediatric center. Data were collected from the medical records of 91 patients who underwent thyroidectomy and attending the Thyroid Diseases Outpatient Clinic, a multidisciplinary service jointly managed by endocrinologists, head and neck surgeons, and pediatric surgeons at Albert Sabin Children's Hospital (HIAS), from January 2013 to May 2023. All patients included in the study were aged 18 years or younger and had undergone partial or total thyroidectomy for benign or malignant thyroid diseases. The analyzed variables included gender, age at the time of surgery, geographic origin, prior and current pathological history, thyroid hormone levels, ultrasound findings, cytological evaluations, and histopathological results. Data were described in a general context, differentiating between benign and malignant cases, followed by a comparative analysis of the two groups. The study was approved by the Ethics Committee under protocol number 70588423.8.0000.5042, adhering to established research guidelines and ethical standards.

### Statistical analysis

Categorical data were expressed as absolute counts and percentages. The chi-square test or Fisher's exact test was used to assess associations between categorical data. Continuous data were first assessed for normality using the Shapiro-Wilk test, the evaluation of histograms, Q-Q plots and dispersion measures. Parametric data was expressed as mean ± SD, and non-parametric data as median and interquartile range. For comparisons between two independent groups, the authors used the Student's *t*-test for parametric data and the Mann-Whitney test for non-parametric data. Logistic Regression was used to assess the risk factors associated with malignancy. The analyses were carried out using SPSS software for Macintosh (Version 23.0. Armonk, NY: IBM Corp).

### Variable selection

To identify the most relevant predictors while minimizing the risk of overfitting due to the limited sample size, the authors applied a backward stepwise selection approach. This method sequentially removes non-significant variables based on a predefined criterion, aiming to retain a parsimonious and stable model. To validate the robustness of this selection strategy, the authors also applied a forward stepwise selection procedure. Both approaches led to the identification of a similar set of predictors, suggesting low collinearity among the variables and supporting the consistency and reliability of the final model.

## Results

### General descriptive analysis

Ninety-one pediatric patients underwent thyroid surgery, of which 68 (74.7 %) were female. This trend related to sex remained the same when a lower age cut-off was used (below 10 years). The average age was 13 years, ranging from 4 to 17 years. Most of the patients came from the countryside of the State (61.5 %). Just two patients (2.2 %) had a history of external beam radiotherapy in the neck due to the treatment of non-Hodgkin's lymphoma and Ewing's sarcoma, and their histologies after thyroidectomy were benign and malignant, respectively. One patient (1.1 %) had a hereditary tumor syndrome associated with differentiated thyroid cancer (Proteus syndrome) and their histopathological report was benign.

Thyroid function tests (TSH levels) were documented in 77 patients, resulting in 63 (81.8 %), 8 (8.8 %) and 6 (7.8 %) cases of euthyroidism, hyperthyroidism and hypothyroidism, respectively. Of the eighth children with hyperthyroidism, five had nodular uptake on scintigraphy and three had diffuse uptake.

According to the ultrasound findings, 62 patients (79.5 %) operated on had unimodular thyroid disease, with an average size of the largest nodule of 2.4 cm. The thyroid nodules were distributed as follows: 45 cases (49.5 %) in the right lobe, 26 cases (28.6 %) in the left lobe, two cases (2.2 %) in the isthmus, and 12 cases (13.2 %) bilaterally. Histopathological analysis revealed that both nodules located in the isthmus were papillary carcinomas. As for the echogenicity of the largest nodule, 32 patients (35.2 %) had hypoechoic nodules, ten (11 %) had isoechoic solid nodules, five (5.5 %) had hyperechoic solid nodules, 16 (17.6 %) had mixed nodules and six (6.6 %) were cystic. The presence of calcifications on ultrasound showed that 18 cases (19.8 %) had nodules with microcalcification, while 53 (58.2 %) had no calcification and only one (1.1 %) had macrocalcification. Of the 18 patients characterized with microcalcification on ultrasound, 17 (94.5 %) had malignant histology.

A total of 69 cytology results were obtained through fine-needle aspiration biopsy (FNAB) and classified according to the Bethesda system as shown in Table 1. Among the patients with a cytological result of Bethesda III, approximately 36 % had histology indicating malignancy, whereas for Bethesta IV, it was 25 %.

**Table 1:** Needle aspiration cytology in children with thyroid nodules.

	Total Group (*n* = 91)	Benign (*n* = 54)	Malignant (*n* = 37)
FNA Result			
Bethesda I	7 (7,7)	5 (9,3)	2 (5,4)
Bethesda II	25 (27,5)	22 (40,7)	3 (8,1)
Bethesda III	11 (12,1)	7 (13)	4 (10,8)
Bethesda IV	4 (4,4)	3 (5,6)	1 (2,7)
Bethesda V	12 (13,2)	1 (1,9)	11 (29,7)
Bethesda VI	10 (11)	0 (0)	10 (27)
Not perfomed or not found	22 (24,2)	16 (29,6)	6 (16,2)

Categorical data expressed as absolute count and percentages in parentheses.

There were 51 partial thyroidectomies (56 %) and 40 total thyroidectomies (44 %) performed. The indications included large unimodular or multinodular goiters (42.8 %), hyperthyroidism due to Graves' disease (3.3 %), hyperthyroidism caused by toxic nodular disease (5.5 %), nodules suspicious for cancer (45 %), and prophylactic thyroidectomy (3.3 %). Three patients underwent prophylactic thyroidectomy: two with a genetic diagnosis of multiple endocrine neoplasia type 2A (MEN 2A) and one with Proteus syndrome associated with multinodular goiter. Completion thyroidectomy was required in nine patients initially undergoing partial thyroidectomy due to the diagnosis of malignancy on histopathological examination.

### Descriptive analysis - benign histology subgroup

Fifty-four patients had benign histopathologic findings, with a mean age of 13 ± 3 years, and a predominance of females (72.2 %). Of the total, only six (11 %) had any previous pathological disease. TSH levels were normal in 35 (64.8 %) cases, six (11.1 %) were low and four (7.4 %) were high, nine (16.7 %) had no records. Seven children underwent scintigraphy, with three patients showing diffuse uptake and four with nodular uptake.

Ultrasound revealed that 37 (68.5 %) patients had only one nodule. The average size of the largest nodule was 2.7 cm (2.2; 3.6). Regarding location, the distribution was 26 (48.1 %), 15 (27.8 %), 0 (0 %), 9 (16.7 %) in the right lobe, left lobe, isthmus and bilateral, respectively. As for the echogenicity of the largest nodule, 13 (24.1 %) patients had a hypoechoic nodule, five (9.3 %) solid isoechoic nodules, two (3.7 %) solid hyperechoic nodules, 16 (29.6 %) mixed nodules and five (9.3 %) were cystic. Only one patient with benign histology had microcalcification on ultrasound.

Partial thyroidectomy (lobectomy with isthmectomy) was performed in 42 patients (77.8 %), with the primary indications being unimodular bulky goiter (73.8 %), nodules with Bethesda III or IV cytology (16.6 %), and toxic nodular hyperthyroidism (9.5 %). Total thyroidectomy was performed in 12 children (22 %). The indications included Graves' disease (three cases); two cases with Bethesda category III cytology; one case with Bethesda category IV and one case with Bethesda category V; two cases with large multinodular goiter and Bethesda category II; three cases of prophylactic thyroidectomy.

### Descriptive analysis - malignant subgroup

Thirty-seven patients (40.6 %) were diagnosed with malignant nodules, with a mean age of 13 ± 4 years. The youngest patient was four years old. Females constituted the majority of the sample, accounting for 78.4 % (23 patients). Eleven (29.7 %) children presented at the first appointment with metastatic cervical lymphnodes. Euthyroid patients accounted for 28 cases (75.7 %), while patients with elevated or decreased TSH levels were both two cases each (5.4 %). In five cases (13.5 %), TSH values were not documented. Scintigraphy was performed in one patient, revealing nodular uptake and the histological analysis confirmed follicular carcinoma.

The ultrasound data showed that 26 (70 %) patients had only one nodule in the thyroid gland. The average size of the largest nodule was 2.21 cm, with a range of 0.69 to 4.6 cm. The most commonly affected location was the right lobe, accounting for 19 cases (51.4 %), followed by the left lobe with 11 cases (29.7 %), the isthmus with two cases (5.4 %), and bilateral involvement in three cases (8.1 %). Regarding echogenicity, hypoechoic solid nodules were observed in 51.4 % of cases, isoechoic solid nodules in 13.5 %, hyperechoic solid nodules in 8.1 %, and cystic nodules in 2.7 %. None of the patients operated on for cancer had mixed nodules. No macrocalcifications were identified in thyroid nodules among these patients, while microcalcifications were present in 17 cases (45.9 %), no calcifications in 14 cases (37.8 %), and calcification data were unavailable for six cases (16.2 %).

All patients with malignant histology underwent total thyroidectomy, including nine cases where completion thyroidectomy was performed following an initial partial thyroidectomy. These patients were diagnosed with malignancy only after the histopathology reports were finalized. Combined neck dissection was performed in 67.6 % of cases. Among these, 14 cases (37.8 %) involved only level VI dissection ipsilateral to the tumor, nine cases (24.3 %) included levels II to VI ipsilateral dissection, and two cases (5.4 %) included bilateral lateral dissection of levels II to VI. Only two patients who underwent ipsilateral neck dissection of level VI were found to have no metastases.

**Figure 1:**
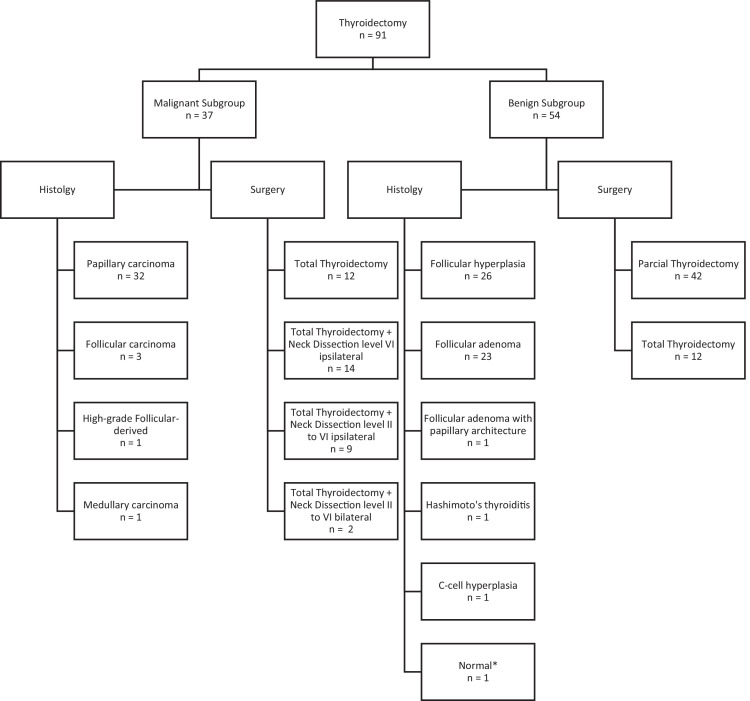
Flow chart summarizing the type of surgery and the histopathological diagnosis.

The most common type of cancer identified was papillary carcinoma (86.7 %) and its subtypes were distributed as follows: classic - 21 cases (65.6 %), classic encapsulated - three cases (9.3 %), follicular infiltrative - three cases (9.3 %), oncocytic - two cases (6.2 %), diffuse sclerosing - one case (3.1 %), tall cell - one case (3.1 %), and solid - one case (3.1 %). For follicular carcinomas, there was one minimally invasive case and two angioinvasive encapsulated cases. The only patient with a high-grade follicular variant had poorly differentiated thyroid carcinoma as the subtype. The type of surgery and the histopathological diagnosis are summarized in Figure 1.

* A patient underwent total thyroidectomy due to a specific mutation in the **RET** gene.

The pathological staging, based on tumor size and spread (TNM 8th Edition), was distributed as follows: T1a - nine cases (24.3 %), T1b - 13 cases (35.1 %), T2 - ten cases (27 %), T3a - two cases (5.4 %), and T3b - three cases (8.1 %). Lymph node staging in this group was: N0 - nine cases (24.3 %), N1a - 14 cases (37.8 %), N1b - 12 cases (32.4 %), and Nx - two cases (5.4 %).

Among patients with papillary thyroid carcinoma, the probability of having persistent cervical disease and/or distant metastases, as classified by the American Thyroid Association, was low in 11 cases (34.3 %), intermediate in 12 cases (37.5 %), and high in nine cases (28.1 %).

### Evaluation of factors associated with the presence of malignant tumors

In a bivariate analysis, the risk factors associated with the presence of malignant tumors were age less than or equal to ten years, hypoechoic solid composition, and the presence of nodules with microcalcifications (Table 2).

**Table 2:** Risk factors associated with the presence of a malignant tumor.

	What was the histopathological result?		
	Benign (*n* = 54)	Malignant (*n* = 37)	p*
Age, years	13 ± 3	13 ± 4	0.492
Age ≤10 years	7 (13)	12 (32,4)	0.025
Sex			0.507
Female	39 (72,2)	29 (78,4)	
Male	15 (27,8)	8 (21,6)	
Thyroid ultrasound			0.967
Even a lump	38 (79,2)	26 (78,8)	
More than one nodule	10 (20,8)	7 (21,2)	
Location			
Left lobe	15 (27,8)	11 (29,7)	0.840
Right lobe	26 (48,1)	19 (51,4)	0.764
Bilateral	9 (16,7)	3 (8,1)	0.347
Isthmus	0 (0)	2 (5,4)	0.163
Nodule size			0.675
< 1 cm	6 (13,3)	6 (16,7)	
> 1 cm	39 (86,7)	30 (83,3)	
Composition of the largest nodule			
Hypereoic solid	2 (3,7)	3 (8,1)	0.393
Isoechoic solid	5 (9,3)	5 (13,5)	0.735
Hypoechoic solid	13 (24,1)	19 (51,4)	**0.007**
Calcifications in the nodule			
Microcalcifications	1 (1,9)	17 (45,9)	**<0.001**

Categorical data expressed as absolute counts and percentages in brackets. Quantitative data expressed as median and interquartile range.

⁎ The Chi-square or Fisher's exact test was used for categorical data, and the Mann-Whitney test for continuous data.

Table 3 shows a logistic regression of independent risk factors for the presence of malignant tumors. The final model indicates that children aged ≤ 10 years and the presence of microcalcifications in nodules of children undergoing thyroidectomy are significantly more likely to have a malignant tumor.

**Table 3:** Logistic regression analysis for risk factors of malignant thyroid tumors in pediatrics.

	Malignant Tumor			
	Initial model		Final model*	
	Odds ratio (95 % CI)	p	Odds ratio (95 % CI)	p
Sex (male)	0357 (0067; 1897)	0.227		
Age ≤ 10 years	5624 (1149; 27,534)	0.033	5,69 (1,21; 26,747)	**0.028**
Nodules on ultrasound (more than one nodule)	0676 (0061; 7537)	0.75		
Location of the nodule (left lobe)	0563 (0,04; 7892)	0.67		
Location of the nodule (right lobe)	0622 (0052; 7372)	0.706		
Nodule size (>1 cm)	1113 (0,19; 6531)	0.906		
Composition of the nodule (Hypoechoic solid)	3792 (1002; 14,35)	0.05	3302 (0915; 11,913)	0.068
Microcalcifications (yes)	34,252 (2951; 397,599)	0.005	28,644 (3291; 249,314)	**0.002**

⁎ The backward stepwise method was used to arrive at the final model with the most important variables.

**Figure 2:**
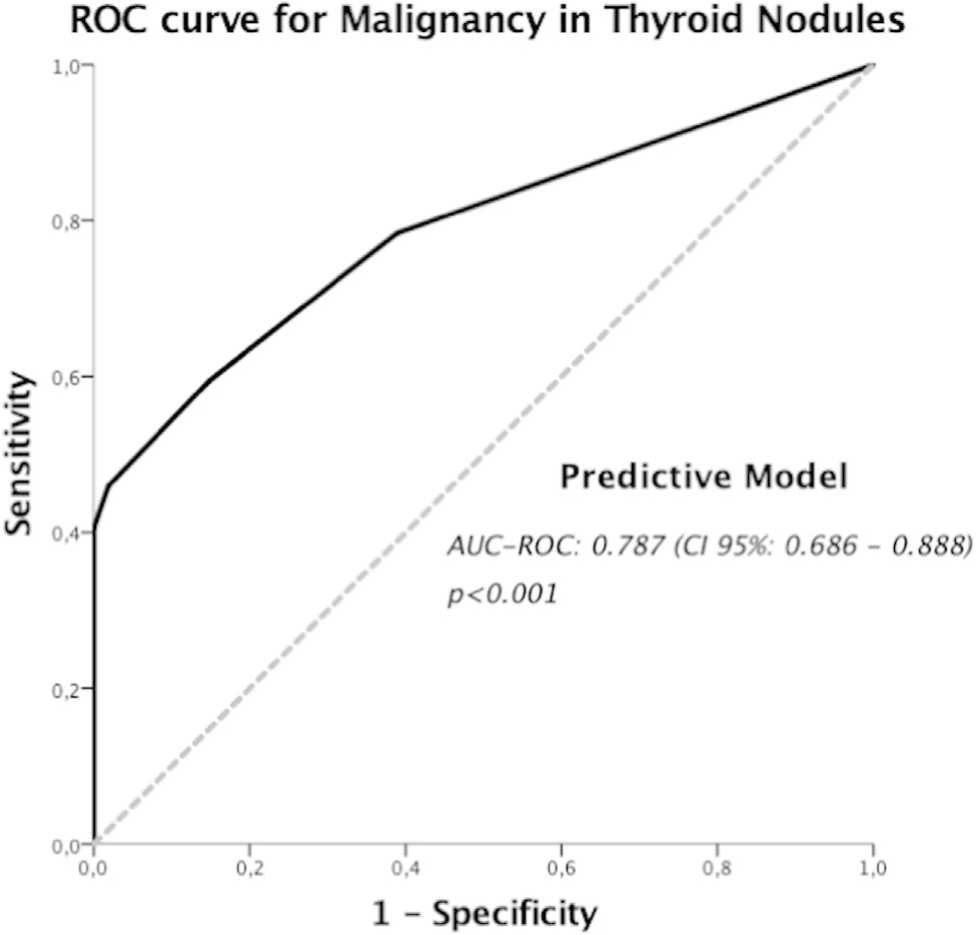
Receiver Operating Characteristic (ROC) curve for the predictive model including age ≤ 10 years, nodule composition, and presence of microcalcifications, for malignancy in thyroid nodules.

The ROC curve for the predictive model including age ≤ 10 years, nodule composition, and microcalcifications demonstrated an area under the curve (AUC) of 0.787 (CI 95: 0.686 – 0.888) (*p* < 0.001), indicating good discriminatory performance. The optimal probability threshold determined by the Youden index was 0.44, which had 59 % of sensitivity and 85,2 % of specificity (Figure 2).

## Discussion

Thyroidectomy is the cornerstone of treatment for malignant thyroid diseases and is also indicated for selected benign conditions, such as symptomatic bulky goiters, Graves' disease, and hyperthyroidism caused by toxic nodular disease.^10^ Although uncommon, thyroid cancers in children have increased in recent years, either due to better surveillance and improved diagnostic tests, or due to other factors, such as environment, diet and lifestyle.^11^,^12^ As a result, there has been a growing interest among professionals in researching and managing this disease in pediatric patients.

In the present study, 91 thyroidectomies were performed, with 51 partial thyroidectomies and 40 total thyroidectomies. After the histological results, nine patients who had undergone partial surgery underwent a complete thyroidectomy. The estimated female-to-male ratio for nodular thyroid disease is 3–4:1.^13^ This ratio was consistent in this study, even when applying an age cut-off below ten years. There was no statistical difference between genders in terms of histological findings of malignancy (*p* = 0.5). The average age observed in the present study was 13 years, slightly lower than other studies, which was 14.5 years.^12^,^13^ The youngest patient was only four years old and was diagnosed with papillary carcinoma with undifferentiated areas of insular pattern. The present results, based on a final multivariate analysis model, revealed that children aged ten or younger who underwent thyroidectomy had a significantly higher likelihood of a cancer diagnosis compared to patients older than this age (OR 5.69, *p* = 0.028).

Although exposure to radiation is considered the main risk factor for the development of thyroid cancer in childhood, especially when this exposure occurs in children under the age of five^14^,^15^ and considering that the institution is a reference center for the treatment of childhood cancer, only two patients had a history of radiotherapy in the cervical region.

TSH levels were within the normal range in 82 % of cases, while elevated levels (hypothyroidism) were observed in 8 % and suppressed levels (hyperthyroidism) in 9 % of cases. Previous studies indicate that the rate of surgical indications for hyperthyroidism ranges from 6 % to 7.7 %.^10^,^16^ Of the eight patients operated on for hyperthyroidism, five had nodular uptake on scintigraphy and only one was diagnosed with minimally invasive follicular carcinoma. Diffuse thyroid uptake was present in three patients, all with benign final histology.

Despite the lack of validation of an ultrasound scoring system to help stratify which patients should undergo FNAB, studies show that ultrasound characteristics of a nodule associated with an increased risk of cancer in adults also convey a higher risk of malignancy in children,^17^,^18^ and the presence of a single nodule, as in adults, increases the likelihood of malignancy.^17^ In the present study, 79.5 % of patients who underwent thyroidectomy had a unimodular goiter on ultrasound. However, this finding was not statistically significant for malignancy when compared to thyroids operated on for multiple nodules (*p* = 0.96). Hypoechoic echogenicity was present in 32 % of the ultrasounds, and after logistic regression of independent factors for the presence of malignancy, the hypoechoic nodule showed a tendency towards significance (*p* = 0.068), suggesting a possible association with malignancy. The presence of microcalcification was found in the examination of 18 children, 17 of which (94.5 %) were diagnosed with malignancy. The sonographic finding of microcalcification was strongly associated with the probable presence of malignancy on histology, with an OR of 28.644 (*p* = 0.002). The location of the nodule was not associated with histological findings overall; however, in the two cases where the nodule was located in the isthmus, histopathology confirmed malignancy.

The interpretation of FNAB cytology using the Bethesda classification system plays a crucial role in the evaluation and surgical decision-making for thyroid nodules in pediatric patients. However, recent studies emphasize the need to refine the interpretation of these findings, particularly due to the higher probability of malignancy associated with indeterminate nodules (Bethesda III) and follicular neoplasms (Bethesda IV), with malignancy rates of 35 % and 58 %, respectively.^2^ In this series, 11 patients underwent surgery following indeterminate cytology (Bethesda III), with a final histological diagnosis of malignancy in four cases (36 %). Additionally, four patients had follicular neoplasia cytology (Bethesda IV), of which one case (25 %) was confirmed as malignant on histology.

The proportion of benign and malignant cases varies significantly across studies on pediatric thyroidectomies.^10^,^13^,^19^ In this cohort, 59,4 % of patients who underwent thyroidectomy had benign histology, while 40,6 % were diagnosed with malignancy. Papillary carcinoma was the most prevalent malignant histological subtype (86.7 %), followed by follicular carcinoma 8.1 %, high-grade follicular-derived 1 (2.7 %) and medullary carcinoma (2.7 %). No cases of anaplastic carcinoma were identified in this series. Central compartment node dissection (level VI) and/or lateral neck dissection (levels II–V) were performed in 67.6 % of cases, emphasizing the need for these procedures to be conducted by surgeons with expertise in the indications and techniques required for optimal management.

The present study had limitations. It is a single-center retrospective study with data collection based on information provided in medical records, thus subject to documentation bias. The lack of reliable recording of postoperative complications, for instance, led us to exclude this information from the study. Additionally, children undergoing thyroidectomy represent a less common group compared to adults, and therefore, further multicenter retrospective and prospective studies are needed to better understand this pathology in pediatric patients.

Thyroid surgery remains the cornerstone for treating both benign and malignant thyroid pathologies. Increasingly, clinicians and surgeons are recognizing the distinctions in the clinical, epidemiological, pathological, and molecular characteristics of benign and malignant thyroid diseases, underscoring the need for specialized training in this area. Furthermore, the adoption of a multidisciplinary approach—integrating pediatricians, pediatric endocrinologists, pediatric surgeons, head and neck surgeons, anesthesiologists, nuclear medicine specialists, and pathologists within institutions dedicated to pediatric care—is essential to ensuring optimal outcomes for children with thyroid disorders.

## Authors’ contributions

Data, analytic methods, and study materials will be made available to other researchers by email.

All authors have approved the final version of this manuscript.

## Funding

Ethical approval. CAAE - 70588423.8.0000.5042

This research received no specific grant from funding agencies in the public, commercial, or not-for-profit sectors.

## Conflicts of interest

The authors declare no conflicts of interest.
